# Factors associated with quality of life trajectories among inpatients treated for alcohol use disorders: A prospective cohort study

**DOI:** 10.1016/j.abrep.2020.100285

**Published:** 2020-05-20

**Authors:** Helle Wessel Andersson, Trond Nordfjærn

**Affiliations:** a Department of Research and Development, Clinic of Substance Use and Addiction Medicine, St. Olavs University Hospital, Trondheim, Norway; b Department of Psychology, Norwegian University of Science and Technology, Trondheim, Norway

**Keywords:** Overall quality of life, Alcohol use disorder, Residential treatment, Patient satisfaction, Mental distress

## Abstract

**Aims:**

The main study purpose was to investigate patient- and treatment-related factors associated with overall quality of life (OQOL) trajectories during and after inpatient alcohol use disorder (AUD) treatment.

**Design:**

A large-scale prospective multicenter cohort study of patients with different substance use disorder (SUD) types who were consecutively admitted for inpatient SUD treatment. Data were obtained at treatment entry (T1), discharge (T2), three months after discharge (T3), and one year after discharge (T4). The inclusion criterion was that the patient be dependent solely on alcohol. OQOL data were collected at all four time points. Independent variables included demographics, mental distress, psychiatric disorders, substance use, treatment history, and patient satisfaction.

**Results:**

Among the 611 patients available, 236 met the AUD inclusion criterion and completed T1 assessments. A linear mixed model showed substantial co-occurrence between higher mental distress and lower OQOL. Higher patient satisfaction with inpatient treatment (T2) was associated with higher trajectories of OQOL, whereas abstinence (T3) was not. There was a substantial increase in OQOL from T1 to T2, which then remained stable during the last two assessment time points.

**Conclusions:**

Routine OQOL screening at treatment entry, and targeting mental distress both during and after inpatient treatment, may be associated with improved OQOL among individuals with AUD. Further research should investigate inpatient treatment factors that contribute to OQOL improvement and those that moderate the relationship between patient satisfaction and OQOL.

## Highlights

• Substantial growth in quality of life during the course of treatment.
• Co-occurrence between mental distress and lower quality of life.
• Higher patient satisfaction associated with higher quality of life trajectories.
• Substance use at follow-up was not associated with quality of life.

## 1 Introduction

Individuals in inpatient treatment for alcohol use disorders (AUD) have a range of treatment needs. In particular, they experience prominent physical, psychological, and social problems (McCallum, Mikocka-Walus, Gaughwin, Andrews, & Turnbull, 2016). These factors are important for daily functioning and are profoundly relevant to reintegration into the community (Laudet, 2011). Quality of life, which generally refers to perceptions of well-being across different domains of functioning (Laudet, 2011), has received attention within the addiction treatment field during the past decades (Luquiens, Reynaud, Falissard, & Aubin, 2012). Recent research has also recommended measures of patients’ quality of life as outcome indicators of substance use disorder (SUD) treatment (Laudet, 2011, Picci et al., 2014). Measures of generic or overall quality of life (OQOL), as opposed to health-related quality of life, explore patients’ perceptions (i.e. within physical, mental health, and social domains) independent of other health conditions (Luquiens et al., 2012). OQOL may therefore be particularly relevant as a treatment outcome measure among SUD patients (Laudet, 2011, Picci et al., 2014).

The treatment outcomes of SUD patients may be influenced by patient related factors (i.e. clinical and psychological variables) and treatment factors, such as the content and process of treatment (Flora, 2019, Flora and Stalikas, 2012, Zhang et al., 2008). So far, only a few studies have investigated the factors that may influence trajectories in OQOL among SUD patients. Regarding patient-related factors, one study of patients admitted to detoxification found that baseline mental distress predicted changes in OQOL at six-month follow-up (Vederhus, Birkeland, & Clausen, 2016). Another prospective study of hospitalized SUD patients found no association between patients’ baseline psychiatric symptoms and changes in OQOL at follow-up (Pasareanu, Opsal, Vederhus, Kristensen, & Clausen, 2015). These two studies (using the same OQOL instrument), also reported inconclusive results regarding the role of gender. Pasareanu et al. (2015) reported that compared with males, females had larger improvement in OQOL scores during SUD treatment, whereas Vederhus et al. (2016) did not find an association between gender and OQOL. The influence of SUD patients’ substance use on OQOL is also not well understood. It has been suggested that greater levels of polysubstance use are associated with lower OQOL (Kelly et al., 2018, Lubman et al., 2016). Conversely, reduced alcohol consumption may be associated with significant increases in OQOL (Frischknecht, Sabo, & Mann, 2013). Vederhus and colleagues (2016) reported that abstinence was associated with improved OQOL, while Pasareanu et al. (2015) did not find such an association.

Associations between treatment-related factors and OQOL outcomes have been the subject of only few previous studies. Patient satisfaction measures are recognized as an important tool for evaluating whether treatment factors contributes to improvements (Doyle, Lennox, & Bell, 2013). Higher patient satisfaction with different aspects of inpatient SUD treatment is suggested to be related to perceived benefit of treatment (Andersson et al., 2017, Zhang et al., 2008) and to predict lower alcohol problem severity one year after treatment initiation (Kendra, Weingardt, Cucciare, & Timko, 2015). Although the studies have mainly been confined to patients with mental disorders, there is also evidence that patient satisfaction with treatment (Berghofer et al., 2001), and perceived quality of services is associated with OQOL (Fleury et al., 2018, Holloway and Carson, 2002).

The few available results on factors associated with changes in OQOL among SUD patients are inconclusive. Moreover, previous studies have generally paid little attention to the influence of treatment-related factors on OQOL trajectories among SUD patients. To the best of our knowledge, no studies have investigated OQOL among patients with AUD in SUD treatment and the patient- and treatment-related factors that may influence OQOL trajectories in this patient population. Previous work has also been limited by measuring OQOL at two assessment time points and using statistical methods that do not account for the clustered nature of the data (e.g. the same patients nested over time). In contrast, a multiple OQOL follow-up allows a mixed model examination of trajectories during and after inpatient treatment.

Therefore, the overall study purpose was to investigate patient- and treatment-related factors associated with OQOL trajectories during and after inpatient AUD treatment. Specifically, based on the literature on factors associated with treatment outcome among patients with substance use and mental health issues, we hypothesized: 1) that higher mental distress would be associated with lower trajectories of OQOL and 2) that higher patient satisfaction with treatment and services received would be associated with higher OQOL trajectories.

## 2 Materials and methods

### 2.1 Design and setting

The current study was part of a larger prospective cohort study of patients consecutively admitted for inpatient SUD treatment in Central Norway from September 2014 to December 2016. The study sites were the five largest publicly funded SUD treatment centers in central Norway, providing treatment for different SUD types. Three of these centers offer short-term inpatient treatment (2–4 months) and two provide inpatient treatment > 6 months. Patients undergo ≤ 14-day detoxification prior to intake, if necessary. All the five centers provide comprehensive treatment and recovery programs, focusing on individually based social, biological, and mental health needs through a combination of group and individual therapies.

Research assistants at these units approached patients 1–2 weeks after inpatient admission. In accordance with the Declaration of Helsinki, all patients gave informed consent prior to inclusion. Patients who chose to participate signed a consent form giving explicit permission for researchers to obtain information from their medical records and to reestablish contact for follow-up interviews. The patients filled in questionnaires at treatment entry (T1) and at discharge (T2). Follow-up interviews were conducted by telephone three months after discharge (T3) and one year after discharge (T4). The Regional Committee for Medical Research Ethics in Norway approved the study (application #2013/1733).

### 2.2 Participants

The inclusion criterion was a sole AUD (ICD-10, F10); in cases where a SUD diagnosis was missing (n = 7), the most frequently used drug prior to admission was alcohol. Thus, the exclusion criterion was an illicit drug use disorder (ICD-10, F11-F19).

### 2.3 Data collection and variables

Variables were collected using self-report instruments and medical records. Patient-related variables were selected based on previously reported associations with OQOL (Colpaert et al., 2013, Fleury et al., 2018, Frischknecht et al., 2013, Pasareanu et al., 2015, Vederhus et al., 2016, Zhang et al., 2008). We also included treatment related factors (e.g. satisfaction with treatment, perceived service quality at follow-up), which have been under-investigated as variables associated with OQOL.

### 2.4 OQOL

OQOL was measured at each time point (T1–T4) with the global subscale (QoL-5) (Muller, Skurtveit, & Clausen, 2016) of the QoL-10 (Lindholt, Ventegodt, & Henneberg, 2002). This instrument has been extensively validated and correlates with other established generic quality of life measures, such as the WHOQOL-BREF (Muller et al., 2016). The five items in QoL-5 cover a broad spectrum of quality of life dimensions: physical health; psychological health; relation to self; relation to friends; and relation to partner. Responses to each use a five-point Likert scale from 1 (very good) to 5 (very poor). The raw scores were transformed to a decimal scale, ranging from 0.1 (worst score) to 0.9 (best score) (Vederhus et al., 2016, Ventegodt et al., 2003). The mean Cronbach’s alpha (α) was 0.73 (range 0.65–0.78).

### 2.5 Patient satisfaction and perceived service quality at follow-up

Patients’ satisfaction with treatment was reported at T2. This nine-item instrument was derived from the Patient Experiences Questionnaire for Interdisciplinary Treatment for Substance Dependence (PEQ-ITSD) (Haugum, Iversen, Bjertnaes, & Lindahl, 2017). One additional item from the Treatment Perception Questionnaire (TPQ) (Marsden et al., 2000) was included to obtain patients’ perceptions of time in treatment (“Have you had enough time in treatment to sort out your problems”). A project team of experienced clinicians and researchers selected the items used in the current study based on relevance and utility criteria. Responses to the 10 items were recorded on a five-point Likert scale, ranging from 1 (not at all) to 5 (to a very large degree) (α = 0.86). The average score was used as a patient satisfaction index.

Four items were included to measure perceived service quality at T3. These items reflected whether patients perceived that they had easy access to services, whether the services had helped them make recovery progress, and the degree of user involvement and satisfaction with the outlined plans for further follow-up (α = 0.80). The instrument was scored on a four-point scale from 1 (not at all) to 4 (to a large degree). The average score was used as a perceived service quality follow-up index.

### 2.6 Mental distress and psychiatric disorders

Mental distress was measured at all four time points (T1–T4) using the self-reported Hopkins Symptom Checklist-10 (HSCL-10) (Derogatis, Lipman, Rickels, Uhlenhuth, & Covi, 1974). The Norwegian translation of this 10-item instrument has shown feasible psychometrics (Strand, Dalgard, Tambs, & Rognerud, 2003). Patients reported how frequently they had experienced symptoms related to depression and anxiety during the past seven days on a scale ranging from 1 (not at all) to 4 (extremely) (α = 0.89, range 0.87–0.91); the mean score was used in analyses.

Comorbid psychiatric diagnosis (yes/no) was based on a medical record of any ICD-10 diagnosis (F20–F99).

### 2.7 Substance use and treatment history

Medical records were used for substance use and treatment history information. SUD diagnoses (F0–F19) were classified according to the International Classification of Diseases, 10th revision (ICD-10) (WHO, 1992) Additional substance use information included most frequently used drug type during the six months preadmission. Treatment history included information about any previous inpatient SUD treatment stay (yes/no), length of current stay, and treatment completion/dropout. The patients’ onset age was recorded at T1 with the question: “How old were you the first time you used substances?” Abstinence (yes/no) at T3 was based on the question “Have you used substances for the last four weeks?”

### 2.8 Demographics

Demographic information (e.g. age at intake, gender) was obtained from medical records.

### 2.9 Statistical analysis

Descriptive statistics, including Chi-square test were used to describe sample characteristics. Cohen’s *d* and Cramer’s *V* were used to determine group difference effect sizes for the continuous and categorical measures, respectively. SPSS version 25 was used for these analyses.

Linear mixed modeling was used to investigate patient- and treatment-related factors associated with OQOL trajectories during and after inpatient treatment using Stata 14.2. This modelling approach allows use of all available data including those patients who have missing data on one or multiple assessment time points. Our base model examined both linear and quadratic temporal trends by incorporating Time and Time^2^ as random effects. This decision was based on a visual screening of individual OQOL trajectories, reflecting that respondents differed substantially in both T1 OQOL and trajectories. In the next step, a model tested which patient- and treatment-related factors had fixed effects. Treatment site was also included as a fixed effect, as too few patients were nested in each site to estimate a random effect. Since mental distress was measured on all four assessment points, this variable was entered as a time-varying covariate accounting for variation in mental distress across the entire study period. Both models were tested with both random intercepts and slopes, unstructured covariance matrix, and maximum likelihood (ML) estimation. Inclusion of a random intercept accounts for individual baseline differences in OQOL and random slopes allows for variation in individual OQOL trajectories over time (e.g. improved, declined or unchanged OQOL).

A planned post hoc test of marginal effects with Bonferroni correction examined specific differences in OQOL by Time, adjusting for the remaining factors in the mixed model. A variation inflation factor (VIF) < 4.00 was used as a cutoff for the presence or absence of collinearity (Miles & Shevlin, 2001). A sensitivity analysis was conducted excluding patients who did not participate at all assessment time points (n = 114) and those with incomplete OQOL follow-up data (n = 19).

## 3 Results

### 3.1 Sample

**Fig. A1:**
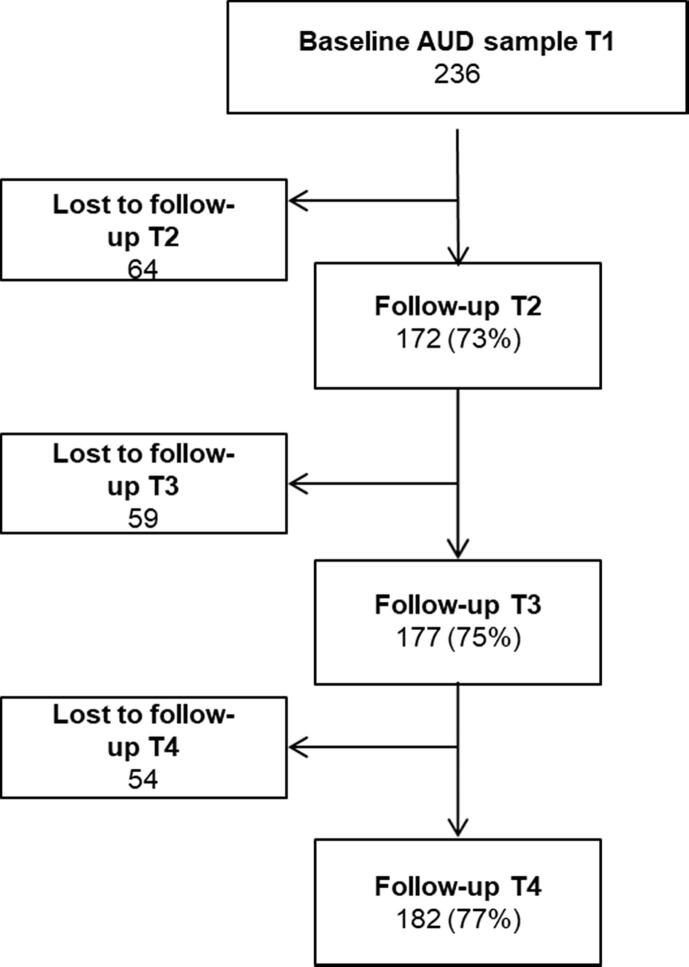
Flowchart of study sample.

T1 assessments were conducted with 611 of 728 eligible patients (84%), of whom 236 satisfied the inclusion criterion of misusing only alcohol. Of the 236 participants at T1, 172 provided data at T2, 177 at T3, and 182 at T4 (see flowchart of study participants in Appendix Fig. A1). In total, 122 patients participated at all assessment time points. Loss to follow-up at T2 was mainly due to treatment dropout (n = 22) or administrative failure (n = 14); attrition at T3 and T4 was because participants did not reply to research assistants’ telephone calls. Table 1 presents study variables and sample characteristics at each assessment time point.

**Table 1:** Sample characteristics^1^ at each assessment time point.

Variables		Baselinesample T1(n = 236)			Respondents at follow-up T2 (n = 172)		Respondents at follow-up T3 (n = 177)		Respondents at follow-up T4 (n = 182)
		n	M (SD) or percent	n	M (SD) or percent	n	M (SD) or percent	n	M (SD) or percent
Age at intake		235	49.12 (11.61)	171	49.76 (11.38)	176	49.87 (11.21)	181	49.58 (11.14)
Onset age		229	15.54 (4.10)	168	15.57 (4.60)	172	15.69 (4.52)	177	15.33 (2.60)
Gender	- Female	73	31.1%	54	31.4%	55	31.3%	59	32.6%
	- Male	162	68.9%	118	68.6%	121	68.8%	122	67.4%
Previous inpatient stay	- Yes	148	62.7%	107	62.2%	113	63.8%	116	63.7%
	- No	88	37.3%	65	37.8%	64	36.2%	66	36.3%
Psychiatric diagnosis	- Yes	67	28.4%	50	29.1%	54	30.5%	57	31.3%
	- No	169	71.6%	122	70.9%	123	69.5%	125	68.7%
Length of stay		236	67.72 (39.95)	172	71.94 (38.04)	177	66.92 (33.50)	182	70.41 (42.36)
Mental distress		236	2.00 (0.73)	171	1.59 (0.49)	177	1.83 (0.71)	181	1.76 (0.72)
OQOL		222	0.57 (0.16)	165	0.68 (0.11)	170	0.63 (0.15)	169	0.64 (0.16)
Patient satisfaction index (T2)				172	4.03 (0.55)	140	4.04 (0.57)	138	4.03 (0.54)
Abstinent (T3)	- Yes					86	48.6%	78	49.7%
	- No					91	51.4%	79	50.3%
Perceived service quality index (T3)						174	3.17 (0.81)	154	3.19 (0.82)

1 Comparison of those with incomplete follow-up data and those who participated at all time points showed that they were similar on all T1characteristics, including OQOL and mental distress. Patients with incomplete follow-up data were somewhat less satisfied with services received at 3-month follow-up (*p* = 0.036) and less likely to report being abstinent at 3-month follow (*p* = 0.002).

Improved OQOL was reported among 63% of the sample at T4, whereas 31% and 6% reported reduced or unchanged OQOL, respectively.

### 3.2 Patient satisfaction

Patients were generally satisfied with the inpatient treatment they received. The aspects of treatment receiving the highest ratings were staff perceptions, staff understanding the type of problem, and availability of staff counseling. Activities offered and time in treatment received relatively lower ratings. Table 2 presents means and variance for each patient satisfaction item.

**Table 2:** Items measuring patient satisfaction at discharge.

Items	N	Mean	SD
Availability of staff counseling	172	4.09	0.72
Have benefited from treatment	171	4.25	0.75
Problems understood by staff	172	4.26	0.72
Opportunities to affect treatment plan	171	3.78	0.92
Felt safe at the institution^a^	171	171	4.54
Satisfactory activities were offered	170	3.86	0.85
Personnel cooperated with next of kin^b^	136	3.83	0.83
Had been prepared for the time after discharge	169	3.89	0.79
Enough time in treatment to sort out problems	171	3.86	0.95
Overall treatment was satisfactory	172	4.21	0.68

a Item excluded from further analyses due to high proportion of respondents (60%) answering in the most positive response category.

b Item excluded from further analyses due to high proportion (21%) of missing responses.

Patients were also generally satisfied with the follow-up services (Table 3). Specifically, involvement in making plans for follow-up and access to services were ranked highest, whereas perceived benefit of follow-up services was rated lowest.

**Table 3:** Items measuring perceived service quality at follow-up (T3).

Items	N	Mean	SD
Have had easy access to follow-up services	174	3.24	0.96
Have benefited from follow-up services	167	2.92	1.13
Have been involved in service needs decisions	160	3.16	0.94
Have had opportunities to affect plans for follow-up	162	3.40	0.86

Note. Items measured on a four-point scale (1 = not at all, 4 = to a large degree).

### 3.3 Prediction of quality of life trajectories

To investigate potential heterogeneity in OQOL trajectories, a base model (Table 4: Model 1) was tested including linear and quadratic temporal trends as random effects. The model showed substantial differences both in T1 OQOL status (intercept, σ = 0.07, p < .000) and slope (σ = 0.001, p < .046). Since this variance warranted further exploration, we tested the full model, including patient- and treatment-related factors as fixed effects.

**Table 4:** Linear mixed model predicting OQOL.

	Model 1				Model 2			
Parameter	Estimate	z-value	*p*-value	95% CI	Estimate	z-value	*p*-value	95% CI
Intercept	0.630	84.15	0.000	0.615; 0.644	0.680	10.34	0.000	0.551; 0.809
Time								
T1 (ref)	–	–	–	–	–	–	–	–
T2					0.562	4.85	0.000	0.033; 0.079
T3					0.040	3.54	0.000	0.018; 0.062
T4					0.034	2.61	0.009	0.083; 0.059
Psychiatric diagnosis (yes)					0.006	0.42	0.676	–0.023; 0.035
Gender (female)					0.020	1.44	0.149	–0.007; 0.047
Age					0.000	0.30	0.762	–0.001; 0.001
Previous inpatient stay (yes)					–0.018	–1.38	0.167	–0.044; 0.008
Abstinent T3 (yes)					0.010	0.80	0.426	–0.015; 0.035
Mental distress					–0.147	–17.99	0.000	–0.163; –0.131
Onset age					0.002	1.47	0.141	–0.001; 0.004
Length of stay					0.000	0.74	0.458	–0.000; 0.001
Patient satisfaction (T2)					0.032	2.60	0.009	0.008; 0.056
Perceived service quality (T3)					0.002	0.21	0.837	–0.015; 0.019
Treatment site					–0.002	–0.41	0.683	–0.012; 0.008
Variance components								
Intercept	0.074	3.46	0.000	–	0.017	1.45	0.073	–
Time	0.033	2.06	0.019	–	0.012	1.25	0.105	–
Time^2^	0.001	1.68	0.046	–	0.001	1.53	0.063	–

As shown in Table 4 (Model 2), high mental distress was strongly associated with reduced OQOL at all four time points. Higher patient satisfaction at T2 predicted higher OQOL growth trajectories. Growth in T2–T4 OQOL trajectories was also substantial compared with T1.

VIF varied from 1.084 to 2.855, indicating that multicollinearity was absent from the mixed model.

**Fig. 1:**
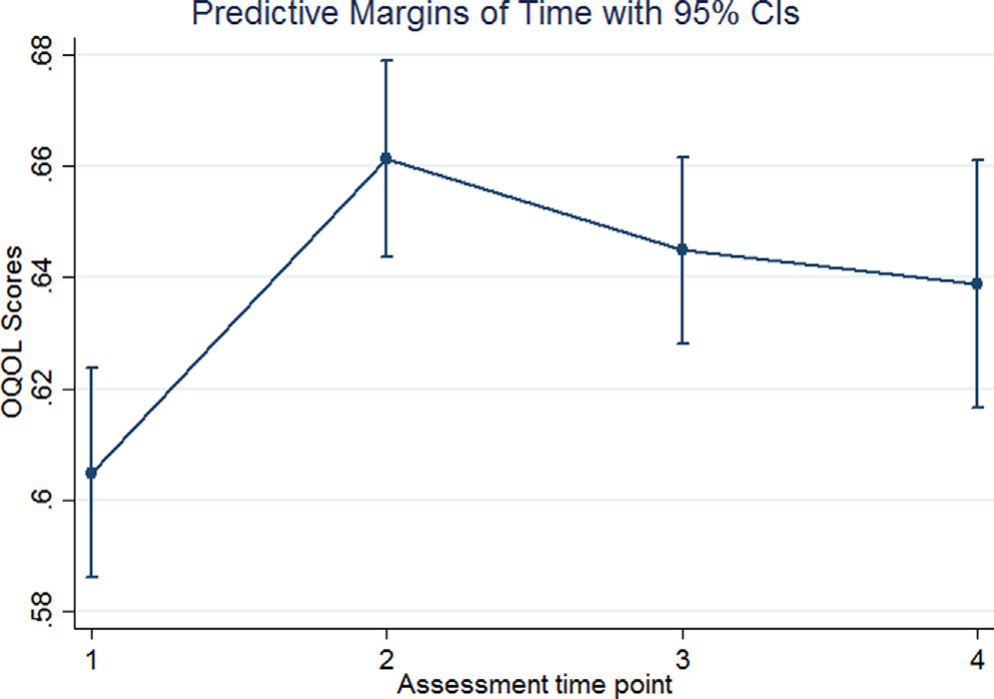
Estimated marginal effects of OQOL by time points (T1–T4).

Fig. 1 shows that the most substantial growth increase was between T1 and T2 (p < 0.000). Growth was weaker at T3 (p = 0.004) and T4 (p = 0.041). Estimated marginal means showed that the growth differences between T2, T3, and T4 did not reach significance.

### 3.4 Sensitivity analysis

Sensitivity analysis across the four assessment time points, excluding those lost to follow-up, essentially reflected similar results as in Table 4 (Model 2). For instance, higher mental distress was strongly associated (z = –18.29, 95% CI = –0.171; –0.138, p < .000) with lower OQOL throughout the study period. Higher patient satisfaction at T2 was also positively associated with OQOL (z = 2.59, 95% CI = 0.008; 0.059, p = .015). Similar time trends in OQOL as those reported in Table 4 (Model 2) and Fig. 1 were detected in the sensitivity analysis, with slightly weaker z-values. Furthermore, female gender (z = 1.97, 95% CI = 0.001; 0.055, p < .049) and older age of onset (z = 2.43, 95% CI = 0.001; p = .010) were weakly associated with higher OQOL in the sensitivity analysis.

## 4 Discussion

The current study investigated patient- and treatment-related factors associated with OQOL trajectories during and after inpatient AUD treatment.

As hypothesized, and in line with previous research among SUD inpatients (Vederhus et al., 2016), the current study showed that higher mental distress was associated with lower OQOL trajectories. The association between mental distress and OQOL trajectories among patients in SUD treatment has been sparsely investigated, and the current study is the first to address this issue among inpatients with AUD. Mental health and general quality of life may be interrelated dimensions. As such, SUD treatment providers may consider incorporating routine OQOL and mental distress screenings at treatment entry, to target patient groups among whom these dimensions should be a focus. Such initiatives, both during and after inpatient treatment, may contribute to more successful treatment outcomes among many patients.

Also as hypothesized, increased patient satisfaction with inpatient treatment was associated with higher OQOL trajectories. This is the first prospective study showing an association between patient satisfaction and OQOL among patients in SUD treatment. The current finding is in line with studies that have reported associations between patient satisfaction with SUD treatment and treatment outcomes, such as perceived benefit of treatment (Andersson et al., 2017) and drug use improvements (Zhang et al., 2008). The result is also congruent with research on patients with mental health problems (Berghofer et al., 2001). Patient satisfaction within substance use treatment may be strongly associated with client engagement indicators and involvement in therapy (Dearing, Barrick, Dermen, & Walitzer, 2005) and may even be a proxy for therapeutic alliance (Simpson, 2004).

Patient perception of follow-up service quality was not significantly associated with OQOL. The importance of consistency and continued care following inpatient treatment is widely acknowledged (Karriker-Jaffe et al., 2018, Manning et al., 2017). Although previous research in this area is scarce, findings among service users with mental disorders have suggested that quality of life is associated with greater service continuity and satisfaction with the help received (Fleury et al., 2018, Holloway and Carson, 2002). The current finding may be related to the low symptom severity of the current sample (as reflected by their relatively high mean QoL-5 follow-up scores), and consequently reduced needs for ancillary support services following inpatient treatment compared with those with more severe illicit drug use and severe mental health problems.

Abstinence three months after discharge from inpatient treatment was not associated with OQOL. This finding contradicts studies emphasizing the importance of abstinence for improving quality of life after SUD treatment (Laudet, 2007, Vederhus et al., 2016). Diverging results may relate to differences in symptom severity between samples. Inconsistent results may also be due to assessment timing and number, type of statistical analyses, and adjustment for other variables. Nevertheless, the current findings are consistent with those of Pasareanu et al. (2015) and with research suggesting limited congruity between abstinence and subjective well-being (Wilson, Bravo, Pearson, & Witkiewitz, 2016). For many who seek treatment for alcohol problems, the treatment goal may be reduced intake rather than abstinence (DeMartini et al., 2014). It should also be noted that abstinence from substances might not have an immediate, positive impact on OQOL. Patients may experience abstinence symptoms in the presence of specific situations and triggers (Marlatt & Gordon, 1985), which could negatively influence OQOL. Longitudinal studies with longer follow-up measurements should elucidate the role of post-treatment abstinence on OQOL.

Most patients in this study (63%) reported improved quality of life at follow-up. These results are consistent with previous research suggesting improved quality of life during the course of SUD treatment (Muller et al., 2017, Pasareanu et al., 2015, Ugochukwu et al., 2013). The current findings showed a growth in OQOL from treatment entry to discharge. Thereafter, OQOL stabilized at a higher level than initially (i.e. at treatment entry). One possible explanation for the current findings is that inpatient substance use treatment takes a psychosocial approach, focusing on key areas for social reintegration, in addition to providing treatment for other substance abuse problems. The mean one-year follow-up OQOL score among our sample was somewhat higher than the scores recently reported in two six-month follow-up studies with more heterogeneous SUD samples (Pasareanu et al., 2015, Vederhus et al., 2016). This may be due to the longer follow-up interval of the current study. The difference may also indicate a relatively lower symptom severity of the current sample, consistent with research suggesting an association between substance use severity and OQOL (Kelly et al., 2018, Lubman et al., 2016). However, patients in the current sample had a mean QoL-5 score at 12 month follow-up significantly below the mean QoL-5 score of 0.71 reported in non-patient samples (Birkeland, Weimand, Ruud, Høie, & Vederhus, 2017). This may reflect either that the effects of treatment on secondary, nondrinking outcomes may require more than a year (LoCastro et al., 2009), or that there are long-term negative effects of AUD (Kendler et al., 2017, Schuckit, 2009).

## 5 Limitations

The study was conducted among patients with AUD, so these findings might not generalize beyond this clinical population. Although the one-year follow-up response rate was comparable with other studies (Adamson, Sellman, & Frampton, 2009), the number of patients who responded at all four time points was modest. Nonrandomness of those with incomplete follow-up data might be a concern. However, additional analyses showed that those who did not respond at follow-up were similar to the analytic sample on all baseline variables. Nonetheless, some differences were found at the three-month follow-up; those with incomplete follow-up data were less likely to be abstinent and less satisfied with follow-up services received. As such, the associations found between OQOL and these two factors may have been attenuated. Moreover, if a larger patient sample had participated at all time points, we may have had greater statistical power to detect factors significantly associated with OQOL. For example, variables that trended to be associated with OQOL, such as previous inpatient stay and onset age (both reflecting dependence severity) and gender, may have reached statistical significance in a larger sample. However, a major strength of the mixed model approach is that it allows use of all available data, including from participants with incomplete data (Peters et al., 2012, Thabane et al., 2013). A sensitivity analysis excluding those lost to follow-up showed results that were similar to the model which also incorporated patients with missing data on one or more assessment time points.

## 6 Conclusions

This study assessing OQOL in a sample of patients with AUD, who were followed for one year after inpatient treatment, extends our knowledge about factors associated with OQOL. Based on these findings we propose that clinicians routinely screen for OQOL at AUD treatment entry, to identify patients for whom this dimension should be a treatment focus. Targeting mental distress both during and after treatment may also be associated with improved OQOL for persons with AUD. The current study also shows that patient satisfaction with different aspects of SUD inpatient treatment is associated with subsequent OQOL improvements. Future research should more closely investigate which aspects of inpatient treatment contribute to improved quality of life among service users, and other factors that may moderate this relationship. Longer-term posttreatment studies of OQOL development trajectories are also needed to determine whether OQOL eventually stabilizes at a higher level compared with pretreatment, or whether it declines to a similar level over time.

## 7 Role of funding sources

This work was supported by the Norwegian University of Science and Technology (NTNU), Trondheim, Norway, St. Olav’s University Hospital, Trondheim, Norway, and Møre and Romsdal Hospital Trust, Ålesund, Norway. The funding sources did not have any significant influences on data collection, analyses, writing, or the decision to submit the manuscript for publication.

## 8 Contributors

H.W.A. designed the study, wrote the protocol, and undertook the initial analyses. T.N. undertook the final statistical analyses. Both authors wrote the manuscript and have approved the final version.

## Declaration of Competing Interest

The authors declared that there is no conflict of interest.
